# Comparison of two ultra-widefield color-fundus imaging devices for visualization of retinal periphery and microvascular lesions in patients with early diabetic retinopathy

**DOI:** 10.1038/s41598-022-21319-9

**Published:** 2022-10-19

**Authors:** Heiko Stino, Susanna Riessland, Aleksandra Sedova, Felix Datlinger, Stefan Sacu, Ursula Schmidt-Erfurth, Andreas Pollreisz

**Affiliations:** Department of Ophthalmology and Optometry, Medical University of Vienna, Währinger Gürtel 18-20, E8i, 1090 Vienna, Austria

**Keywords:** Retina, Medical imaging, Retinal diseases, Diabetes

## Abstract

Comparison of two ultra-widefield (UWF) color-fundus (CF) imaging devices in diabetic patients for visualization of retinal periphery and detection of early microvascular lesions. The total gradable areas (TGA) seen on non-mydriatic CF-images of two UWF-imaging devices (Optos Daytona P200T; Zeiss Clarus 700) were compared and differences in projected area measured. Retinal periphery outside the 7 standard fields (7SF) was divided into: F3 temporal, F4 superotemporal, F5 inferotemporal, F6 superonasal, F7 inferonasal. DR stage was evaluated in the 7SF and the TGA on images of both devices and compared using Cohens κ. 67 eyes of 67 patients (52.5 ± 15.3 years) were analysed. DR stages in the 7SF were no (n = 36 Optos, n = 35 Clarus), mild (n = 16 Optos, n = 17 Clarus), and moderate DR (n = 15). Optos depicted significantly more area in F3 (median [interquartile range]; 2.41% [1.06–4.11] vs 0% [0–0], *P* < 0.001) and Clarus in F7 (3.29% [0–7.69] vs 0% [0–3.27], *P* = 0.002). In 4 eyes DR-stage was higher using Optos due to peripheral lesions not seen on the Clarus. Interrater reliability of DR-stage on both devices was almost perfect in the 7SF (κ = 0.975) and the TGA (κ = 0.855). Reliability in detecting signs of early DR is high on both devices. Clarus allowed for better visualization of the inferonasal field, Optos of the temporal field.

## Introduction

Diabetic Retinopathy (DR) is still a leading cause of visual impairment or blindness^1^. Since this disease progresses asymptomatically in early stages, nationwide screening programs are essential to detect early pathologies and identify typical lesions to minimize retinal damages^2^. Changes that can be diagnosed during fundus examination are dot and blot hemorrhages, microaneurysms, cotton-wool-spots, and intraretinal microvascular abnormalities (IRMA)^3^.

It has been three decades since the modified Airlie-House-Classification from the Early Treatment Diabetic Retinopathy Study was introduced to determine stages of DR. Here 7 stereoscopic pictures (7 standard fields, 7SF) are used to, when combined, show approximately 34% of the retina to evaluate lesions^4^. The International Clinical Diabetic Retinopathy (ICDR) Severity Scale was proposed in 2003 to simplify DR grading in clinical use^5^.

To date advanced imaging modalities are available allowing visualization up to far peripheral retinal areas. Wide-Field-Imaging (WFI) devices have a field of view (FOV) of 60°–100° and enable displaying the midperiphery. Ultra-Wide-Field-Imaging (UWFI) devices are defined as having a FOV of up to 220°, depicting an area of more than 3 times the size of the 7SF and including the visualization of the far periphery^6^.

Studies suggest that imaging the retinal periphery might have a potential influence on the classification of DR as well as on estimated risk of progression^7–11^.

Furthermore, it has been postulated that agreement was high when comparing diagnosis from non-mydriatic-UWFI-modalities with indirect ophthalmoscopy in mydriasis, allowing a much quicker and still reliable retinal examination^12,13^.

Two of the latest UWFI devices are the Optos Daytona P200T and the Zeiss Clarus 700. Optos Daytona P200T (Daytona, Optos^®^, Dunfermline, Scotland, UK) enables a single 200° image depicting 82% of the retina in less than 0.4 s in miosis. Its software Optos*Advance*™ allows documentation and monitoring as well as analysis including measurement tools. Additionally, several images can be combined to a 220°-montage visualizing 97% of the retina^14^. Another new commercially available device is the Zeiss Clarus 700 (Carl Zeiss Meditec AG, Oberkochen, GER). Different imaging modalities are available for either WFI or UWFI. In the WF-mode a single 133° image is captured in less than 0.2 s. In the UWF-mode two images are combined to create a montage of 200°.

Recent studies have compared Optos P200DTx with Zeiss Clarus 500 and found that both devices were generally consistent in assessing DR severity with the former capturing a significantly larger retinal area^15–17^.

To date there is no direct comparison of Zeiss Clarus 700 and Optos Daytona P200T 200° images regarding differences in FOV and microvascular lesion detection in early stages of diabetic retinopathy.

## Methods

In this prospective, observational, cross-sectional case series we included patients with a confirmed diagnosis of diabetes from the diabetic outpatient unit of the Department of Ophthalmology at the Medical University of Vienna (Austria). The study was performed in accordance with ICH-GCP guidelines. Approval was given from the Medical University of Vienna ethics committee. Written informed consent was obtained from all subjects prior to enrollment in the study. Inclusion criteria were best corrected visual acuity (BCVA) of more than 0.8 decimal and early non proliferative diabetic retinopathy (NPDR) (no to moderate NPDR). Exclusion criteria were myopia and hyperopia of ≥ 3 diopter (dpt), history of previous retinal disease, panretinal photocoagulation, intravitreal injection, trauma, and advanced DR (severe NPDR to proliferative DR).

Non-mydriatic images were taken by trained personnel from our department using the Optos Daytona P200T and the Zeiss Clarus 700. The Optos uses a red (635 nm) and a green (532 nm) laser to scan the retina and reproduce a color image^14^. Instead of a laser the Clarus uses a white LED flash consisting of red (585–640 nm), green (500–585 nm) and blue (435–500 nm) LED and an infrared laser diode (785 nm) creating a so called “true color” fundus image as seen during funduscopy^18^. Patients were asked to open their eyes as widely as possible during examination. Eyelashes were not fixated to simulate a real-world clinical situation. Two images at most were acquired with the Optos, the best of which was used for grading. The final Clarus image consists of two separate images (nasal and temporal), leading to a total of 4 images taken per patient, the best of which were used for analysis. The sequence of image acquisition with the Optos and the Clarus device was alternated between acquisitions.

Image quality was evaluated by a grader assessing vascular structure and was graded as low quality when central sharpness was reduced and/or artifacts such as shadows or flashes were noticeable in the image center.

ICDR-grading was used to determine the stage of DR on the acquired images using a 7SF-grid and the total gradable area (TGA) for both devices. The TGA excluding eyelashes, distortions, and blurriness was measured using the built-in measurement tools on each device. Disease severity levels were classified as proposed by Wilkinson et al.^5^. Mild DR was defined as microaneurysms only. If more than microaneurysms including hemorrhage were present, DR was graded as moderate.

A 7SF-grid was overlaid on Optos images by the Optos*Advance*™ editing software. A reading specialist certified by the Vienna Reading Center (VRC) using a VRC custom software placed the grid with computer-assisted adjustment for size and the disc as a reference point on the Clarus images.

**Figure 1:**
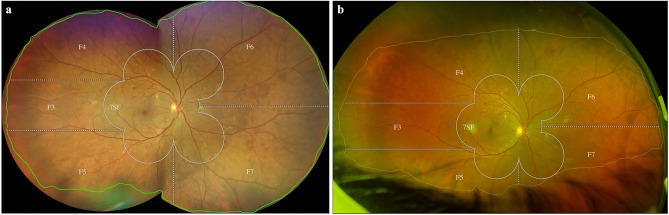
Ultra-widefield images acquired with the Clarus (**a**) and Optos (**b**). The total gradable area which excludes eyelashes, distortions, and blurriness is measured using the measurement tools on each device (**a**: green line, **b**: yellow line). EDTRS 7 standard fields (7SF) and peripheral fields F3-F7 are used for classification of DR. Color differences due to imaging modalities are notable with the Clarus using “true color” due to white LED, while Optos uses a red and green laser. Differences of gradable area can be seen as the Clarus allows further insight into the inferior fields (F5, F7) and the Optos into the temporal field (F3).

Retinal periphery outside the 7SF was divided in five additional peripheral fields as proposed by Silva et al.^10^: temporal (F3), superotemporal (F4), inferotemporal (F5), superonasal (F6), and inferonasal (F7) (Fig. 1).

To evaluate size differences in the gradable area for each peripheral field, side-by-side comparison of images of the same eye acquired with both devices was performed after DR stage was assessed. Anatomical landmarks were used to determine which areas were visible on images of one but not of the other device. These areas were then measured in mm^2^ using the built-in measurement software of the respective device. To more accurately compare the area obtained by these two imaging modalities and reduce distortions due to minor measurement differences with the devices’ softwares, we used percentage (Difference = mm^2^/TGA of same device × 100) instead of mm^2^. This represents the percentage of the TGA not seen on the other device in each peripheral field.

Differences in detection of hemorrhages were evaluated for each field and were specified as one or more than one hemorrhage difference with each device.

Statistical calculations were performed using IBM SPSS Statistics (Version 27.0. Armonk, NY: IBM Corp.). The agreement of DR-stage between the two devices and the 7SF and the TGA on each device was calculated using a Cohens κ. Interpretation was based on Landis and Koch who defined 0.0–0.2, 0.21–0.40, 0.41–0.60, 0.61–0.80, and 0.81–1.00 as slight, fair, moderate, substantial, and almost perfect agreement^19^. Additional 95% Confidence Intervals (CI) were calculated. Differences of the gradable area detected in the peripheral fields F3–F7 compared to the other device was calculated using a Wilcoxon signed-rank test. Bonferroni correction was performed equaling α = 0.0083 for a desired α = 0.05.

## Results

Sixty-seven eyes of 67 patients (63 right and 4 left eyes) were imaged with both Optos Daytona P200T and Zeiss Clarus 700 at the same study visit. Twenty-five patients (37%) were female. Mean age ± standard deviation (SD) was 52.5 ± 15.3 (range 18–77) years. Mean best corrected visual acuity (BCVA; Snellen) in decimal was 1.09 ± 0.17 (range 0.63–1.25). Twenty-two (33%), 41 (61%), 3 (4.5%), and 1 (1.5%) patients had diabetes type 1, type 2, late onset autoimmune diabetes in the adult (LADA), or maturity onset diabetes of the young (MODY), respectively. Mean duration of diabetes was 13.3 ± 10.3 (range 0–47) years.

Differences in peripheral fields in percentage of the TGA and in mm^2^ as measured on each device and number of patients with different stages of DR detected with both devices in the 7SF and the TGA are listed in Table 1.

**Table 1:** Measurements for Clarus and Optos device.

	Clarus	Optos
TGA in mm^2^ (mean ± SD)	701.54 ± 112.22	562.6 ± 90.85
**Differences in peripheral fields: (median, IQR)**		
F3 in % [mm^2^]	0 (0–0)	2.41 (1.06–4.11)
	[0 (0–0)]	[15 (5–24)]
F4 in % [mm^2^]	2.71 (0–4.54)	1.88 (0–3.39)
	[19.44 (0–34.71)]	[11 (0–23)]
F5 in % [mm^2^]	1.77 (0–5.04)	0.65 (0–2.74)
	[12.37 (0–38.9)]	[3 (0–15)]
F6 in % [mm^2^]	1.94 (0–4.81)	1.84 (0–5.01)
	[13.16 (0–31.62)]	[10 (0–30)]
F7 in % [mm^2^]	3.29 (0–7.69)	0 (0–3.27)
	[26.07 (0–58.25)]	[0 (0–18)]
**Stage of DR in 7SF (n = eyes)**		
No DR	35	36
Mild DR	17	16
Moderate DR	15	15
**Stage of DR in TGA (n = eyes)**		
No DR	35	34
Mild DR	16	14
Moderate DR	16	19

*7SF* 7 standard fields, *DR* diabetic retinopathy, *IQR* interquartile range, *SD* standard deviation, *TGA* total gradable area.

TGA is the area measured excluding eyelashes or distortions. Difference in the peripheral fields F3–F7 corresponds to the area not seen on the other device due to eyelashes or distortions observed through comparison of anatomical landmarks and is given in percentage of the TGA and in mm^2^ as measured on both devices.

**Figure 2:**
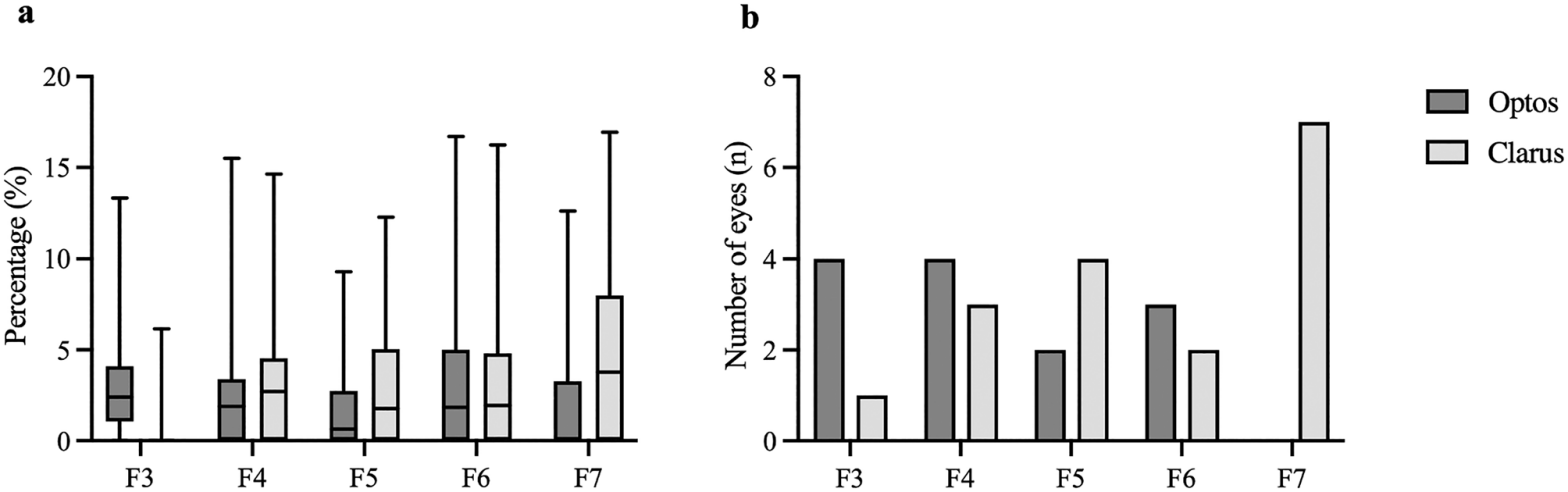
(**a**) The percentage of total gradable area (TGA) not seen on the other device in the peripheral fields F3–F7 evaluated via side-by-side comparison of images of the same eye acquired by both devices is visualized using box plots. The inferior boundary of the box indicates the 25th percentile, a black line within the box marks the median, and the superior boundary of the box indicates the 75th percentile. Whiskers extend to the smallest/largest value within the interval [25th percentile − 1.5 × interquartile range; 75th percentile + 1.5 × interquartile range]. (**b**) Number of eyes with one or more than one hemorrhages detected in the area not seen on the other device in the peripheral fields F3–F7.

There was a significant difference in F3 (*P* < 0.001) and F7 (*P* = 0.005) with the Optos instrument having a significantly larger gradable area in F3, while the Clarus device was superior in F7 (Fig. 2). Images obtained by Clarus showed less artifacts due to eyelashes in the inferior fields compared to images taken by the Optos instrument.

When comparing the areas not visible on the other device due to a smaller FOV or overlapping eyelashes in the peripheral fields, we found additional hemorrhages on images of both devices. A higher number of eyes with additional hemorrhages were detected in F3 using the Optos device, compared to a higher number in F7 using the Clarus instrument (Fig. 2). In F3, we observed 2 patients with one hemorrhage, as well as 2 patients with more than one hemorrhage with the Optos that were not detected when using the Clarus. In F7 we found in 5 patients one hemorrhage, in 2 patients more than one which were not detectable using the Optos.

Ten (15%) images acquired with Clarus and 3 (4%) obtained with Optos were classified to be of low quality (*P* = 0.051).

Comparing DR stages between 7SF and TGA on Optos images by expert graders revealed an almost perfect interrater reliability of κ = 0.854 (p < 0.001). The larger TGA resulted in worse DR stages in 6 eyes (9%) with 4 classified as moderate instead of mild and 2 as mild instead of no DR in the 7SF.

With the Clarus, interrater reliability between DR stages as evaluated in the 7SF and the TGA was almost perfect with κ = 0.976 (p < 0.001). The larger TGA achieved by the Clarus resulted in 1 patient being diagnosed with moderate instead of mild DR in the 7SF.

Exact agreement of DR stage in the 7SF results and the TGA was reached in 93% (n = 62 eyes) with the Optos and 99% (n = 66 eyes) with the Clarus. An agreement within 1 level of DR-stage was achieved in 100% (n = 67 eyes). On both devices examination of the TGA resulted in a higher stage of DR diagnosed in some patients due to hemorrhages in the periphery that were not visible in the 7SF.

The interrater reliability regarding stages of DR between both devices was almost perfect for the 7SF (κ = 0.975, p < 0.001) and the TGA (κ = 0.855, p < 0.001), respectively. Comparing DR stages on 7SF and TGA images between both devices, exact agreement was reached in 99% (n = 66 eyes) on 7SF and 93% (n = 62 eyes) on TGA images. One level DR stage agreement was reached in 100% of eyes on the 7SF and the TGA. One eye of a 42-year-old female patient with a different stage of DR detected in the 7SF was graded mild instead of no DR on the Clarus image. This was due to a MA not seen on the Optos.

**Figure 3:**
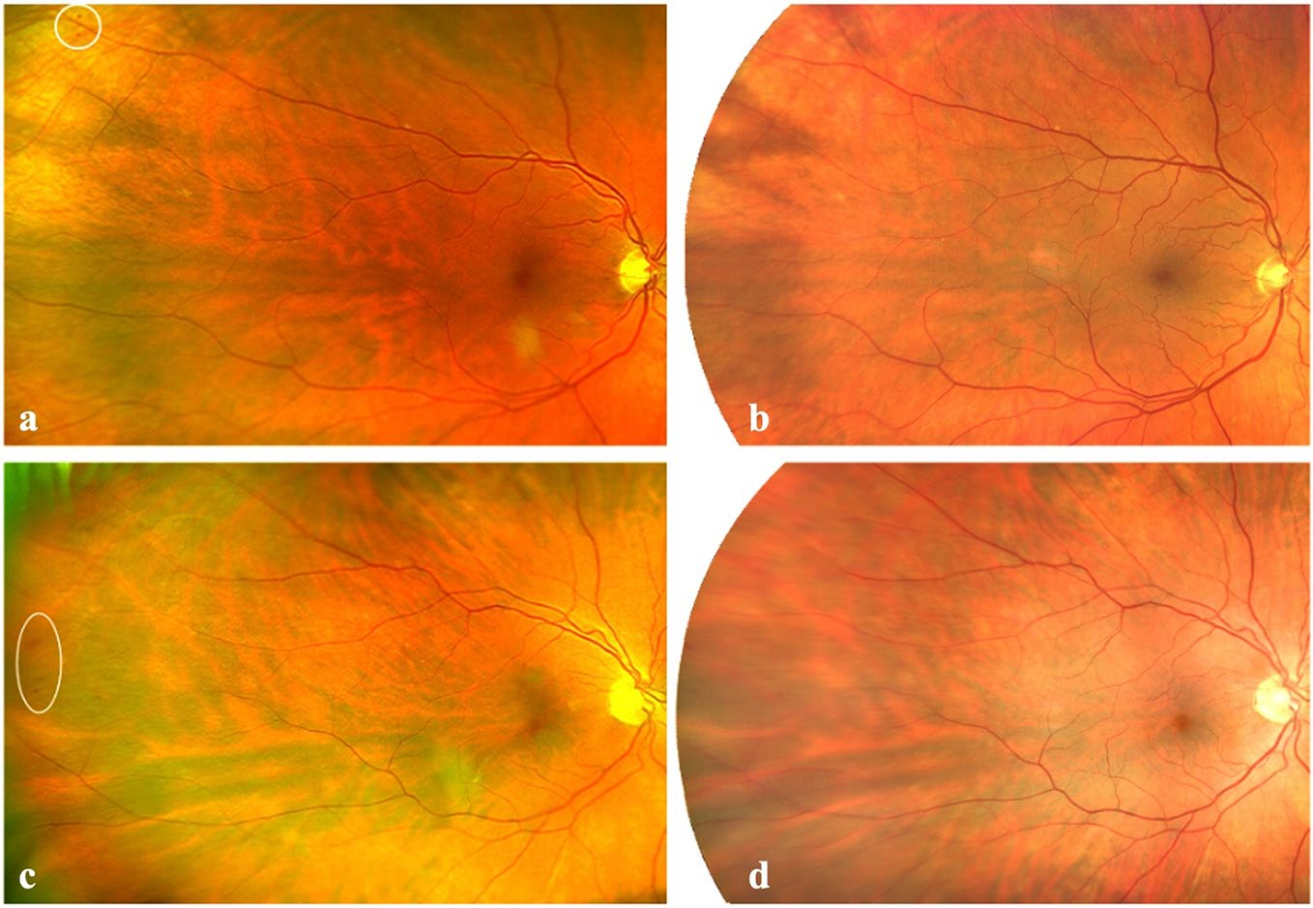
Optos images of two right eyes (**a**,**c**) of two patients with peripheral temporal lesions highlighted with white circles outside the field of view of the corresponding Clarus images shown in (**b**) and (**d**).

Six eyes were graded differently on both devices based on the examination of the TGA. One eye was graded 1 DR level higher with the Clarus. This was the 42-year-old female patient described above. 5 eyes were graded one level higher with the Optos device. Of those 5 eyes, 4 were graded higher due to hemorrhages (n = 2) and MAs (n = 2) in the periphery not depicted on the Clarus images (Fig. 3). One was graded higher because of an artifact that looked like a hemorrhage seen on the Optos image not visible on the Clarus image and confirmed by indirect funduscopy.

## Discussion

While Optos depicts more of the peripheral temporal and Clarus more inferonasal retinal area, both devices offer a high reliability in detecting diabetic lesions and assessing early DR stages.

Since DR is still among the leading causes for visual impairment worldwide, efficient screening tools are needed to prevent severe vision loss in underscreened communities^1^. Telemedical approaches or automated detection through artificial intelligence may offer a time- and cost-effective screening method especially for earlier stages of DR to determine whether medical attendance is needed before sight-threatening complications arise^20–23^. Although artificial intelligence has a high sensitivity in detecting diabetic lesions, image quality might be a crucial factor in providing consistent and reliable results^24^. Knowing which device to use to acquire high quality images for analysis is a key factor in maximizing reliability, especially in the assessment of early stages represented by subtle microvascular abnormalities.

Chen et al. compared the TGA captured by previous models of Optos and Zeiss Clarus and observed a significantly larger retinal area depicted on Optos images (765.6 mm^2^ vs 566.5 mm^2^, ratio 1.35:1)^15^. Conti et al. reported similar findings with a larger TGA on the Optos compared to the Zeiss Clarus (781.67 mm^2^ vs 686.03 mm^2^, ratio 1.14:1)^17^. In contrast to those recent studies, we found that the Optos Daytona P200T depicted a smaller TGA of 562.6 ± 90.85 mm^2^ compared to 701.54 ± 112.22 mm^2^ on the Zeiss Clarus 700 (ratio 1:1.25). However, the TGA in this study was measured with the built-in measurement tools of the respective device, which allows no direct comparison of data.

Compared to Chen et al. we did not use four quadrants to describe differences in imaging the periphery but five peripheral fields as proposed by Silva et al.^10,15^. Chen et al. described an advantage of the Optos in all quadrants compared to the Clarus with the largest additional area depicted in the temporal quadrant^15^. Matsui et al. compared retinal vessel branches to determine the effective fields seen on both devices and found more retinal vessels in the superotemporal quadrant on the Optos 200Tx, compared to more in the inferonasal quadrant on the Clarus 500^25^. Using anatomical landmarks to compare images, we could also detect an advantage of the Optos in our temporal peripheral field F3. However, overlapping eyelashes causing a smaller gradable area were more often observed on Optos images leading to an advantage of the Clarus in depicting the inferior field F7. In these additional areas, diabetic hemorrhages were detected. However, a higher stage of DR due to this additional area was only observed in 4 eyes on Optos images.

We found that only 4% of images on the Optos were of poor quality compared to 15% on the Clarus. That might be due to the different methods of obtaining the images by both devices. On Optos, a single image of the retina is obtained, while the Clarus device requires two images that, after montaging, show a field of view of up to 200°. This makes the Clarus more difficult to use for unpracticed operators or patients with lower compliance. Chen et al. described that 25.7% (n = 18) of their Zeiss Clarus images were single capture images due to the technicians’ inability to take multiple images needed for the UWFI montage^15^.

We found that both devices had advantages in depicting different peripheral fields with images obtained by the Optos having a larger gradable area in the temporal peripheral field F3 and by the Clarus in the inferior fields F5 and F7. This resulted in a higher detection rate of diabetic lesions in F3 on the Optos and F7 on the Clarus, but had no significant influence on DR stage on both devices. However, in 4 eyes the better depiction of the temporal periphery led to a higher stage of DR on the Optos compared to the Clarus.

Hirano et al. evaluated κ values for DR stage detected with UWF-Optos and WF-Clarus images of 0.88 in the 7SF and 0.79 in the TGA^16^. Our results support their findings with κ values of κ = 0.977 in the 7SF and κ = 0.864 in the TGA, indicating an almost perfect interrater agreement of both devices. Exact agreement was reached in nearly 99% of eyes in the 7SF and in 91% using the TGA. Agreement within 1 level of DR stage was reached in all images using the 7SF as well as the TGA. The differences in one patient in the 7SF observed on the Optos and Clarus were due to lower quality of the Optos image, resulting in a lower stage of DR due to a MA seen on the Clarus. Khan et al. reported an advantage of the Clarus in identifying lesions of the central retina and of the Optos in detecting lesions in the mid-peripheral and peripheral retina^26^.

Munuera-Gifre et al. analysed the locations of diabetic lesions in 94 patients, postulating that the upper temporal quadrants contained most abnormalities^27^. We found no significant differences in our peripheral temporal upper field F4 comparing both devices, indicating that they cover this area to a similar extent.

Aiello et al. recently described that peripheral lesions may account for increased DR severity by 2 or more steps in 11% of eyes when directly comparing 7SF and UWFI^11^. Rasmussen et al. compared 7SF-images with non-mydriatic WF-images and found a higher DR stage in 16% of eyes due to diabetic lesions in the periphery outside the 7SF. They found an exact agreement with 7SF images in 76% and one-level agreement in 99% of eyes^28^.

Silva et al. compared UWF-100-degree images with the 7SF and found an exact DR severity agreement in 84% and an agreement within 1 level in 91%^12^. Our results are in line with these findings, suggesting that the extra FOV in UWFI allows the detection of lesions in the periphery. Analysis of the TGA resulted in a higher stage of DR in in 7% and in 1% of eyes on Optos and Clarus images, respectively. Nevertheless, we found that differences between DR-stage using 7SF compared to the TGA on both devices were little with an excellent interrater reliability. The Optos showed an exact agreement of DR stage in 93% and the Clarus in 99%. Both had an agreement within 1 level in 100% comparing 7SF with the TGA.

A limitation of our study is the measurement of the TGA with the built-in software of each device allowing no direct inter-device comparison regarding area indicated in mm^2^.

Both the Optos and the Clarus offer a high reliability in detecting signs of early DR with the Optos depicting more peripheral temporal and the Clarus more inferonasal area.

## Data Availability

The datasets used and/or analysed during the current study are available from the corresponding author on reasonable request.

## References

[1] Leasher JL (2016). Global estimates on the number of people blind or visually impaired by diabetic retinopathy: A meta-analysis from 1990 to 2010. Diabetes Care.

[2] Jampol LM, Glassman AR, Sun J (2020). Evaluation and care of patients with diabetic retinopathy. N. Engl. J. Med..

[3] Lois N, McCarter RV, O’Neill C, Medina RJ, Stitt AW (2014). Endothelial progenitor cells in diabetic retinopath. Front. Endocrinol..

[4] Early Treatment Diabetic Retinopathy Study Research Group (1991). Grading diabetic retinopathy from stereoscopic color fundus photographs—an extension of the modified Airlie House classification: ETDRS report number 10. Ophthalmology.

[5] Wilkinson CP (2003). Proposed international clinical diabetic retinopathy and diabetic macular edema disease severity scales. Ophthalmology.

[6] Choudhry N (2019). Classification and guidelines for widefield imaging: Recommendations from the International Widefield Imaging Study Group. Ophthalmol. Retin..

[7] Mackenzie PJ, Russell M, Ma PE, Isbister CM, Maberley DAL (2007). Sensitivity and specificity of the Optos Optomap for detecting peripheral retinal lesions. Retina.

[8] Price LD, Au S, Chong NV (2015). Optomap ultrawide field imaging identifies additional retinal abnormalities in patients with diabetic retinopathy. Clin. Ophthalmol..

[9] Wessel MM, Aaker GD, Parlitsis G, Cho M, D’Amico DJ, Kiss S (2012). Ultra-wide-field angiography improves the detection and classification of diabetic retinopathy. Retina.

[10] Silva PS (2015). Peripheral lesions identified on ultrawide field imaging predict increased risk of diabetic retinopathy progression over 4 years. Ophthalmology.

[11] Aiello LP (2019). Comparison of early treatment diabetic retinopathy study standard 7-field imaging with ultrawide-field imaging for determining severity of diabetic retinopathy. JAMA Ophthalmol..

[12] Silva PS, Cavallerano JD, Sun JK, Noble J, Aiello LM, Aiello LP (2012). Nonmydriatic ultrawide field retinal imaging compared with dilated standard 7-field 35-mm photography and retinal specialist examination for evaluation of diabetic retinopathy. Am. J. Ophthalmol..

[13] Borrelli E (2020). Nonmydriatic widefield retinal imaging with an automatic white LED confocal imaging system compared with dilated ophthalmoscopy in screening for diabetic retinopathy. Acta Diabetol..

[14] Optos webpage. http://www.optos.com (Accessed 22 Sept 2021).

[15] Chen A (2021). Quantitative comparison of fundus images by 2 ultra-widefield fundus cameras. Ophthalmol. Retin..

[16] Hirano T, Imai A, Kasamatsu H, Kakihara S, Toriyama Y, Murata T (2018). Assessment of diabetic retinopathy using two ultra-wide-field fundus imaging systems, the Clarus® and Optos™ systems. BMC Ophthalmol..

[17] Conti TF (2020). Comparison of widefield imaging between confocal laser scanning ophthalmoscopy and broad line fundus imaging in routine clinical practice. Ophthalmic Surg. Lasers Imaging Retina.

[18] Zeiss webpage: http://www.zeiss.com (Accessed 22 Sept 2021).

[19] Landis JR, Koch GG (1977). The measurement of observer agreement for categorical data. Biometrics.

[20] Stanimirovic A (2020). Tele-retina screening of diabetic retinopathy among at-risk populations: An economic analysis. Can. J. Ophthalmol..

[21] Bernd L (2000). Telemedical screening of diabetic retinopathy. Diabetes Care.

[22] DeBuc DC (2016). The role of retinal imaging and portable screening devices in tele-ophthalmology applications for diabetic retinopathy management. Curr. Diab. Rep..

[23] Abràmoff MD (2010). Automated early detection of diabetic retinopathy. Ophthalmology.

[24] Abràmoff MD, Folk JC, Han DP, Walker JD, Williams DF, Russell SR, Massin P, Cochener B, Gain P, Tang L, Lamard M (2013). Automated analysis of retinal images for detection of referable diabetic retinopathy. JAMA Ophthalmol..

[25] Matsui Y (2019). Comparisons of effective fields of two ultra-widefield ophthalmoscopes, Optos 200Tx and Clarus 500. Biomed. Res. Int..

[26] Khan R (2021). Comparison of two ultra-widefield cameras with high image resolution and wider view for identifying diabetic retinopathy lesions. Transl. Vis. Sci. Technol..

[27] Munuera-Gifre E (2020). Analysis of the location of retinal lesions in central retinographies of patients with Type 2 diabetes. Acta Ophthalmol..

[28] Rasmussen ML (2015). Comparison between Early Treatment Diabetic Retinopathy Study 7-field retinal photos and non-mydriatic, mydriatic and mydriatic steered widefield scanning laser ophthalmoscopy for assessment of diabetic retinopathy. J. Diabetes Complicat..

